# RNAspa: a shortest path approach for comparative prediction of the secondary structure of ncRNA molecules

**DOI:** 10.1186/1471-2105-8-366

**Published:** 2007-10-01

**Authors:** Yair Horesh, Tirza Doniger, Shulamit Michaeli, Ron Unger

**Affiliations:** 1 Department of Computer Sciences, Bar-Ilan University, Ramat-Gan 52900, Israel; 2 The Mina & Everard Goodman Faculty of Life Sciences, Bar-Ilan University, Ramat-Gan 52900, Israel

## Abstract

**Background:**

In recent years, RNA molecules that are not translated into proteins (ncRNAs) have drawn a great deal of attention, as they were shown to be involved in many cellular functions. One of the most important computational problems regarding ncRNA is to predict the secondary structure of a molecule from its sequence. In particular, we attempted to predict the secondary structure for a set of unaligned ncRNA molecules that are taken from the same family, and thus presumably have a similar structure.

**Results:**

We developed the RNAspa program, which comparatively predicts the secondary structure for a set of ncRNA molecules in linear time in the number of molecules. We observed that in a list of several hundred suboptimal minimal free energy (MFE) predictions, as provided by the RNAsubopt program of the Vienna package, it is likely that at least one suggested structure would be similar to the true, correct one. The suboptimal solutions of each molecule are represented as a layer of vertices in a graph. The shortest path in this graph is the basis for structural predictions for the molecule. We also show that RNA secondary structures can be compared very rapidly by a simple string Edit-Distance algorithm with a minimal loss of accuracy. We show that this approach allows us to more deeply explore the suboptimal structure space.

**Conclusion:**

The algorithm was tested on three datasets which include several ncRNA families taken from the Rfam database. These datasets allowed for comparison of the algorithm with other methods. In these tests, RNAspa performed better than four other programs.

## Background

Small non-coding RNA (ncRNA) molecules are DNA sequences that are transcribed into RNA but are not translated further into protein sequences; rather they mediate their cellular functions as RNA molecules. These molecules have been the subject of much interest, as recent studies have shown that they are widespread in a variety of organisms, conserved in evolution, and play essential enzymatic and regulatory roles in many cellular processes. Experimentally, these molecules have been difficult to identify because of their small size and generally low abundance. Therefore, they were overlooked for many years both in experimental and bioinformatic studies. It is only in the last few years that systematic genome-wide computational and experimental screens were carried out to identify ncRNA-encoding genes, both in prokaryotes and eukaryotes [1-4]. ncRNA molecules were found to be involved in ribosome RNA maturation and modification (snoRNA), replication (telomerase RNA), protein translocation (SRP RNA), gene silencing (miRNA), and many other functions [5,6]. Furthermore, it was suggested [7,8] that cellular control based on ncRNA is a major determinant of the complexity of organisms, especially in higher eukaryotes, as ncRNA molecules may offer an additional cellular control mechanism that complements protein-based regulation.

Computational identification of ncRNA molecules is more difficult than identifying protein coding genes. On the sequence level, to identify protein coding genes in a genome, one can take advantage of the existence of several characteristics or motifs included in all coding sequences such as open reading frames, the three base periodicity of codons and the amino-acid coding preference. In addition, other signs of genes are embedded in the regions flanking the coding sequence, including promoters, enhancer sequences, and others. In general, similar signals have not been identified, and are clearly weaker for ncRNAs. On the structural level, ncRNA molecules are characterized by a secondary structure that is formed by pairing of complimentary bases (A-U, G-C, and to a lesser extent U-G) along the single strand molecule. However, it is clear that given a small alphabet of four letters with this pairing potential, many secondary structures are possible even for sequences that do not encode ncRNA [9]; thus, it is difficult to recognize ncRNAs solely based on their potential to form secondary structure [10].

Despite these difficulties, many classes of ncRNA have been identified computationally. Techniques such as sequence conservation in concert with thermodynamic stability have been successful in computationally identifying ncRNA molecules [11,12]. However, identifying ncRNA is only part the challenge. As mentioned above, most ncRNA are characterized by distinctive secondary structures, which often correspond to their respective functions. Thus, given an ncRNA sequence, the task of accurately predicting its secondary structure is of utmost importance.

The two main programs that are widely used to predict the structure of single ncRNA molecules are Mfold [13,14], and RNAfold of the Vienna Package [15,16]. Both programs use a dynamic programming approach to calculate the structure with the Minimal Free Energy (MFE) [17]. The basic algorithm is straightforward and was originally suggested by Nussinov [18] to identify the structure with the maximal amount of base pairing. However, the actual implementation for secondary structure prediction of ncRNA molecules is more complicated. Correct free energy calculation involves many parameters that are relevant to the energy function of a folded RNA molecule, including stem energy (the length of the stem and the type of hydrogen bonds that stabilize it), the size of loop structures, stacking energy between consecutive pairs of nucleotides, temperature, etc. [13,19]. While these programs are the result of extensive effort and innovation, they often fail to identify the true structure as the prediction with the MFE. For example, a recent review [20] showed that a prediction based on MFE was able to correctly predict the cloverleaf structure of tRNA in only 30% of cases. In one case, these authors noticed that the correct tRNA structure was ranked 104 in the list of predictions suggested by the RNAsubopt [21] program, which is a variant of the basic prediction algorithm and outputs a list of predicted structures which can be ranked by their MFE. A short computational experiment we performed on our dataset (see Results for more details) indicates that a list of 150 suboptimal structures is likely to contain at least one reasonable prediction. Furthermore, if one ranks the list of predictions by their MFE, there is a correlation between the ranking in the list and the probability of a prediction being correct. However, this correlation is rather weak, and it is therefore not usually possible to choose the best prediction on the basis of its MFE. As will be elaborated on below, our method has the ability to identify the correct prediction from within this list.

The difficulties in predicting the structure of a single RNA molecule led to the idea that structure predictions may be more accurate if they are based on multiple sequences of a single class of molecules. It is well known that secondary structure is conserved within ncRNA families (to a greater extent than sequence conservation among these molecules). Thus, simultaneous prediction of the structure of a set of related ncRNA might reveal the correct common structure for the sequences that comprise a particular family. Furthermore, application of a fast comparative prediction algorithm can result in a tool for detecting novel ncRNA molecules. Such a comparative prediction tool can serve as the core component of a bottom-up clustering algorithm [21-25] By iteratively expanding an initial seed of ncRNA candidates that share a common structure, one can identify additional novel families of ncRNA from within a set of ncRNA candidates.

Several algorithms [21-25] have been suggested to find a common structure when the sequence alignment is given. For example, RNAalifold [21,22,25] combines both thermodynamic stability and sequence covariation by using a dynamic programming approach, in which the energy value that determines the inclusion of a given basepair in the final structure reflects not only the thermodynamic complimentarity value of the basepair, but also the degree of covariance of the nucleotide pair within the set of sequences. In a different version of the problem where the secondary structure of each sequence is either known or predicted, MARNA [26] and RNAforester [27] can be used to take these individual secondary structure assignments and align them to produce a global multiple structural alignment.

When the sequence alignment or the individual structure assignments are not pre-determined, several programs attempt to solve the problem of simultaneously finding the best alignment and a shared secondary structure by assuming a common structure for groups of ncRNA molecules. Some programs, such as Dynalign [28], and SCARNA [29] are designed to align and predict the structure of two molecules, while other programs [9,30-32] are designed to align and predict the structure of a larger dataset. Most of these programs are largely based on some variant of Sankoff's dynamic programming algorithm for simultaneously folding and aligning multiple RNA sequences [33]. Sankoff's algorithm considers all possible folds and all possible alignments of the sequences, so while it is thorough, it is prohibitively slow and memory consuming [34]. In order to achieve a reasonable run-time, constrained versions of the algorithm have been implemented. For example, the StemLoc algorithm [32,35] essentially uses Stochastic Context-Free Grammar (SCFG) as a scoring scheme for Sankoff's algorithm. StemLoc has a pre-folding and pre-aligning step which folds each sequence individually and aligns them in pairs based on sequence alone, before implementing the pairwise SCFG stage. Pmcomp [9] is another variant of Sankoff's algorithm, which takes pre-computed basepair probability matrices as input from McCaskill's algorithm [36], and performs pairwise alignments of RNA sequences. PmMulti [9] is a wrapper program that does progressive multiple alignments by repeatedly calling pmcomp. FoldAlignM [37] is a wrapper for the Foldalign program, which is largely based on the pmcomp program.

Sankoff-based algorithms attempt to simultaneously align the sequences and predict their structure. In a deviation from this concept, RNAcast [38,39] bypasses the need to find a global alignment and focuses on predicting the best secondary structure for each sequence. This is done by predicting 'shapes', which are structures at different levels of abstraction, for each sequence, and then finding a consensus shape that is common to all sequences. For each sequence, the final prediction is the structure with the lowest MFE among all the structures that are mapped to that consensus shape.

Our algorithm shares the principle and logic of RNAcast in that it looks for structure predictions for each sequence and searches for them from within a list of suboptimal predictions. However, there is a significant difference between our approach and that of RNAcast. RNAcast depends on the existence of a common 'shape' and assigns these shapes to the same key in a hash table. RNAspa, while operating under the assumption that the sequences have similar structure, does not depend on this assumption and will look for the best path between the predictions even if they are dissimilar. Therefore, as will be shown later, RNAspa is able to produce structural predictions even when the data is 'contaminated' with a small numbers of unrelated molecules.

## Results

Three main problems arise when trying to evaluate the performance of RNA secondary structure prediction algorithms. One is establishing the data set(s) on which to evaluate the performance of the algorithm, the second is identifying 'the real structure' against which the predictions are tested and the final issue is choosing the measures to be used when evaluating a comparison between a structure prediction and the 'real structure'.

### Data Sets

One major problem in the field of RNA structure prediction is the lack of a good common benchmark that can be used to judge the quality of structural predictions. The Bralibase [40] was the first useful resource to address this need. However, Bralibase I offers structural information for only small number of single (not families of) sequences. Bralibase II, which offers aligned sets of five large families, concentrates on the alignment aspects of the problem; namely, it shows how RNA sequences should be aligned relative to each other, but it does not supply a secondary structure for each sequence. Bralibase 2.1 [41] includes a larger set of aligned sequences, but again it does not include a structural assignment for each sequence. Thus, it is not suitable for testing the validity of our algorithm, which returns a structure prediction for each molecule.

As in many other similar studies (i.e. [26,35,42-44]), Rfam [45] was used as the source for the dataset in this study. Rfam version 7.0 [46] is a large collection of ncRNA families. The seed alignment of each Rfam family contains known representative members of the family as well as a structural assignment, which was hand-curated.

#### First data set

To enable comparison with previous studies, we used the same families that were used by Hamada et al. [31]. The dataset includes eight ncRNA families: tRNA, Tymovirus/Pomovirus tRNA-like 3' UTR element (Tymo), Purine riboswitch (Purine), Lysine riboswitch (Lysine), SAM riboswitch (S box leader) (SAM), FMN riboswitch (RFN element) (FMN), Cobalamin riboswitch (Cobalamin), and glmS glucosamine-6-phosphate activated ribozyme (glmS).

These RNA families form a diverse dataset including tRNA and tRNA-like molecules, riboswitches and ribozymes. tRNA molecules have a highly conserved structure from bacteria to man and function in protein translation. The Tymo RNAs are similar to tRNA molecules in structure and adenylation at the 3' end, but unlike tRNA are not aminoacylated. They are present in the 3' UTR of viral RNAs, and their structure is important for viral replication. Riboswitches are composed of a single metabolite-binding aptamer and a single expression platform that function together to regulate genes in response to changing metabolite concentrations. Ribozymes are RNAs with the ability to act as enzymes and cleave target RNA.

These RNA families have well-characterized biological functions and possess distinct RNA secondary structures. The number of stems of the family members varies from three (Purine riboswitch) to nine (Cobalamin) and their length varies from 66 (tRNA) to 373 (Cobalamin) bps. The average standard deviation of lengths within each family as well as the average sequence identity is detailed in Table 1. For comparison with other currently used programs, we randomly selected groups of ten sequences from each family, so that each sequence was selected at most once and so the number of sequences for each family would not exceed 50. All the other sequence groups used for testing various aspects of RNAspa's performance were selected with the same criteria. The eight families with their structural annotations are available [Additional files 1, 2, 3].

**Table 1:** Comparison of RNAspa's accuracy versus that of other prediction programs

Family	Num. of seed seq. in Rfam	Num. of datasets of ten	Avg. sequence identity	Avg. length and STD	MCC					
					
					RNA- subopt's first choice	RNA- spa	Stem- Loc	pmMulti	Fold-AlignM	RNAcast
tRNA	1114	5	43%	71 ± 4	0.64	**0.86**	0.61	0.73	0.95	0.45 (3)
Tymo	28	2	68%	79 ± 3	0.76	**0.93**	0.98	0.72	0.94	0.92 (1)
SAM	71	5	62%	111 ± 15	0.59	**0.66**	0.38	0.64	0.53	0.62 (1)
Lysine	60	5	50%	179 ± 7	0.63	**0.68**	0.28	0.64	0.52 (1)	0.71 (1)
Purine	37	3	56%	96 ± 1	0.68	**0.86**	0.51	0.74	0.78	0.72 (1)
Cobalamin	171	5	46%	200 ± 27	0.38	**0.40**	0.12	no data	no data	0.41
FMN	48	4	60%	135 ± 19	0.32	**0.44**	0.25	0.45	0.64	0.47 (1)
glmS	14	1	51%	159 ± 40	0.58	**0.61**	0.38	no data	no data	no data

Each family was represented by several non-overlapping datasets (see third column). The MCC score is averaged among the datasets of each family. The number of datasets in which the program failed to produce an output appears in brackets. "no data" denotes that the program failed on all datasets. In some cases, StemLoc returns a consensus structure for only part of a dataset. Therefore, the MCC score reported here only reflects those sequences for which a structure was predicted. See [Additional File 5] for raw data.

#### Second data set

As RNAcast (see below) is the most similar algorithm to RNAspa, we also directly compared RNAspa against RNAcast using the same dataset on which RNAcast was evaluated [38]. This dataset contains the following families: the transfer tRNA; the lin4 miRNA; the 5S ribosomal RNA; the signal recognition particle SRP RNA; IRES: the viral internal ribosome entry sites element; the Purine riboswitch and the SAM riboswitch; the small nuclear RNAs: U1, U2 and U12.

#### Third data set

This dataset contains 5 randomly chosen SSU (small subunit) rRNA sequences (accession numbers: X59604.1 U23936.1, U07367.1, M54937.1, M19172.1). Their sequences and structures were downloaded from [47]. These sequences range in length from about 1700 to 2000 bps. These sequences with their structural annotations are available [Additional File 3].

### The structural standards

Unlike protein structures, where there is a large set (over 30,000 entries) of three dimensional structures determined by the highly accurate experimental methods of X-ray crystallography and NMR, experimental data for both secondary and tertiary structure of RNA molecules is very scarce. X-ray structures are available for only a few molecules. For other molecules, cross-linking data and other biochemical studies can provide some information on their secondary structure. For most families of ncRNA, the structural information, as provided by Rfam and other databases, is based on computational methods such as Infernal [48-50], and thus is not sufficiently reliable as a source for structural 'standards'. For seven of the eight families that constitute the data set used in this study, the secondary structure information provided by Rfam is cited as coming from published experimental studies. Technically, the secondary structural information in Rfam is encoded by the color-coding of complementary regions that forms the stems in each structure, which is equivalent to a string representation of the structure in bracket notation.

### Measures of accuracy

To evaluate and compare the predictions provided by RNAspa with the 'correct' structure, we used the Matthews Correlation Coefficient (MCC) which correlates, on a base by base basis, between the prediction and the correct structure. The MCC measure is used in many studies to evaluate the accuracy of RNA secondary structure prediction methods [31,34,42,51,52]. After counting the number of True-Positives (TP), True-Negatives (TN), False-Positives (FP), and False-Negatives (FN) for each alignment relative to Rfam, MCC provides a measure of accuracy, which is expressed as a number ranging from -1 for assignments that are false, around 0 for random assignments, and 1 for assignments that are all true. Note that the single value provided encapsulates all four accuracy measures, and an MCC measure closer to 1 denotes higher accuracy.

MCC=TP⋅TN−FP⋅FN(TP+FP)⋅(TP+FN)⋅(TN+FP)⋅(TN+FN).
 MathType@MTEF@5@5@+=feaafiart1ev1aaatCvAUfKttLearuWrP9MDH5MBPbIqV92AaeXatLxBI9gBaebbnrfifHhDYfgasaacH8akY=wiFfYdH8Gipec8Eeeu0xXdbba9frFj0=OqFfea0dXdd9vqai=hGuQ8kuc9pgc9s8qqaq=dirpe0xb9q8qiLsFr0=vr0=vr0dc8meaabaqaciaacaGaaeqabaqabeGadaaakeaacqWGnbqtcqWGdbWqcqWGdbWqcqGH9aqpdaWcaaqaaiabdsfaujabdcfaqjabgwSixlabdsfaujabd6eaojabgkHiTiabdAeagjabdcfaqjabgwSixlabdAeagjabd6eaobqaamaakaaabaWaaeWaaeaacqWGubavcqWGqbaucqGHRaWkcqWGgbGrcqWGqbauaiaawIcacaGLPaaacqGHflY1daqadaqaaiabdsfaujabdcfaqjabgUcaRiabdAeagjabd6eaobGaayjkaiaawMcaaiabgwSixpaabmaabaGaemivaqLaemOta4Kaey4kaSIaemOrayKaemiuaafacaGLOaGaayzkaaGaeyyXIC9aaeWaaeaacqWGubavcqWGobGtcqGHRaWkcqWGgbGrcqWGobGtaiaawIcacaGLPaaaaSqabaaaaOGaeiOla4caaa@638F@

The four True/False/Positive/Negative parameters were counted, similar to [34] as follows: In the case of left and right brackets at the exact same position, we add 2 to TP. If RNAspa agrees with the Rfam annotation that there is no stem at a certain base, we add 1 to TN. If RNAspa predicts a basepair while Rfam determines that both bases do not participate in any pair, we add 2 to FP, and conversely, if Rfam indicates that two bases form a pair and RNAspa predicted neither, we add 2 to FN. Finally, RNAspa and Rfam may agree that a base participates in a base-pairing, but disagree as to the identity of the binding partner. In this case, both FP and FN are incremented by 1.

### The rank of the 'correct' structures within the list of suboptimal structures

**Figure 1:**
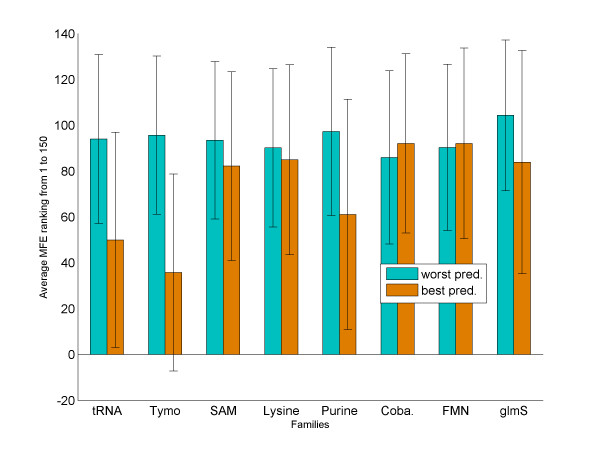
**Average ranking of the best/worst structure prediction**. The average MFE rank of the predicted structure with the best and worst MCC scores. On average, the rank of the best structure (orange) is better (lower) than that of the worst structure (cyan). However, the standard deviation of these averages is very large.

We present here a polynomial time algorithm and its implementation, which performs well for ncRNA structure prediction. The algorithm builds on the ability of the Vienna Package's RNAsubopt to include at least one structure close to the 'true' structure in the set of suboptimal solutions that it suggests. Thus, it is important to first evaluate the improvement that our method offers compared with directly using the results of RNAsubopt. First we analyzed, for our dataset, the accuracy of the predictions of RNAsubopt. RNAsubopt has two modes of operation: 'complete enumeration' ('-e range' option) that enumerates all structures within a predefined range of the MFE structure and 'Boltzmann sampling' ('-p n' option) that samples the space of suboptimal structures following a Boltzmann distribution of their MFE. Unless mentioned otherwise, RNAsubopt was run in the 'complete enumeration' mode in the experiments described below. A comparison between these two modes is shown in Table 2. The 'complete enumeration' mode performed better on shorter sequences and the 'Boltzmann sampling' mode performed better on longer sequences. This can be explained by the nature of these two modes. The two modes trade density for diversity. Within a given energy range in the complete enumeration mode, shorter sequences yield a diverse set of suboptimal structures, but as the length increases an increasing number of suboptimal structures with only minor differences are suggested and therefore sampling becomes more important. We ranked the 150 structures predicted by RNAsubopt for each sequence in two ways: by their MCC score (as compared to the Rfam standard), and by their MFE (as assigned by RNAsubopt). Then, we looked at the structure with the best/worst MCC score and found its position in the MFE ranked list. Averaged results for each dataset are shown in Figure 1. The results show that, on average, the structures with the best MCC scores tend to originate from predictions with better MFEs than those with worse MCC scores. However, these values have very large standard deviations, and thus the correlation is not reliable. Note further that among all the datasets the average MFE-based ranking for the structure with the best MCC score was equal to or greater than 30. Thus, the MFE alone cannot identify the 'correct' formations, and we resorted to a comparative approach.

**Table 2:** Comparing the performance of RNAspa in 'Complete Enumeration' and 'Boltzmann Sampling' modes

Family	Avg. length and STD	Complete Enumeration		Boltzmann Sampling	
		
		Time	MCC	Time	MCC
tRNA	71 ± 4	6.24	0.86	6.51	0.84
Tymo	79 ± 3	6.22	0.93	7.14	0.92
SAM	111 ± 15	14.7	0.66	14.6	0.72
Lysine	179 ± 7	48.53	0.68	45.7	0.72
Purine	96 ± 1	9.53	0.86	9.47	0.84
Cobalamin	200 ± 27	70.25	0.40	71.12	0.43
FMN	135 ± 19	30.08	0.44	25.73	0.49
glmS	159 ± 40	38.79	0.61	33.62	0.64

The time columns indicate the average run-time in seconds per sequence for the corresponding modes of running RNAsubopt. The 'complete enumeration' mode performed better on shorter sequences (tRNA, Tymo, Purine) and the 'Boltzmann sampling' mode performed better on longer sequences (SAM, Cobalamin, FMN, glmS). See [Additional File 6] for raw data.

### Algorithm outline

**Figure 2:**
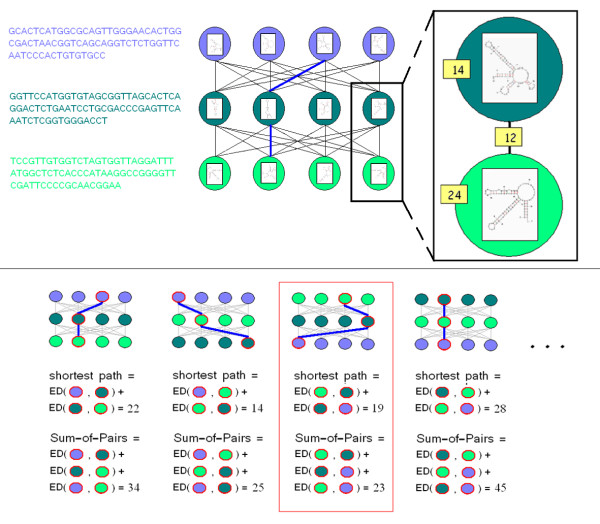
**Schematic view of the data structure used by RNAspa**. Top: Each node carries a suboptimal secondary structure of the sequence belonging to that layer. In a top-down traversal, each node is assigned the shortest path from the top layer to itself. Next, the node with the lowest score on the lowest level is found, and the shortest path is retrieved. Bottom: The process of finding the shortest path reiterates several times. Each time, a different order permutation of the sequences is used. For each shortest path, a Sum-of-Pairs score is calculated. The shortest path with the best Sum-of-Pairs score is returned. In the illustration above, the third shortest path, which is not the shortest of the four paths, is returned because it has the best Sum-of-Pairs.

The algorithm we present here, RNAspa (RNA Shortest Path Approach), aims to predict the secondary structure of a set of ncRNA molecules using a novel approach. The algorithm is presented schematically in Figure 2. The input for our algorithm is a set of N unaligned sequences: *S*_1_, *S*_2_, *S*_3_,..., *S*_*N*_. The first stage uses the Vienna Package RNAsubopt program [21] to suggest a large number of possible structures for each sequence in the set. At the end of the first stage, each *S*_*i *_sequence has V predicted structures: Si1,Si2,Si3,...,SiV
 MathType@MTEF@5@5@+=feaafiart1ev1aaatCvAUfKttLearuWrP9MDH5MBPbIqV92AaeXatLxBI9gBaebbnrfifHhDYfgasaacH8akY=wiFfYdH8Gipec8Eeeu0xXdbba9frFj0=OqFfea0dXdd9vqai=hGuQ8kuc9pgc9s8qqaq=dirpe0xb9q8qiLsFr0=vr0=vr0dc8meaabaqaciaacaGaaeqabaqabeGadaaakeaacqWGtbWudaqhaaWcbaGaemyAaKgabaGaeGymaedaaOGaeiilaWIaem4uam1aa0baaSqaaiabdMgaPbqaaiabikdaYaaakiabcYcaSiabdofatnaaDaaaleaacqWGPbqAaeaacqaIZaWmaaGccqGGSaalcqGGUaGlcqGGUaGlcqGGUaGlcqGGSaalcqWGtbWudaqhaaWcbaGaemyAaKgabaGaemOvayfaaaaa@41DD@ including the optimal structure and V-1 suboptimal structures. In the second stage, each Sij
 MathType@MTEF@5@5@+=feaafiart1ev1aaatCvAUfKttLearuWrP9MDH5MBPbIqV92AaeXatLxBI9gBaebbnrfifHhDYfgasaacH8akY=wiFfYdH8Gipec8Eeeu0xXdbba9frFj0=OqFfea0dXdd9vqai=hGuQ8kuc9pgc9s8qqaq=dirpe0xb9q8qiLsFr0=vr0=vr0dc8meaabaqaciaacaGaaeqabaqabeGadaaakeaacqWGtbWudaqhaaWcbaGaemyAaKgabaGaemOAaOgaaaaa@30C0@ is assigned as a vertex in a graph. Conceptually, the V alternative structures of each sequence *S*_*i *_form a layer of vertices in the graph, and the layers are piled one on top of the other (the V vertices of *S*_*i *_form the layer above the V vertices of *S*_*i*+1_). Each of the vertices belonging to *S*_*i *_is connected by a directed weighted edge to the vertices of *S*_*i*+1_, which is the adjacent layer below. The weight assignment for each edge is calculated based on the similarity between the two alternative structures that it connects. At the end of the second stage, the construction of the graph is completed (see Figure 2 (top)). The resulting graph is a Directed Acyclic Graph (DAG). In the third stage, we compute the shortest path in the graph by a breadth-first traversal from the top (the vertices of the first sequence) to bottom (the vertices of the last sequence) in a linear time in the number of edges. We consider a path in the graph to be the sum of its component edges. The shorter a path is in this graph, the greater is the similarity between the vertices forming the path. In other words, the shortest path represents a set of predicted structures, one predicted structure for each sequence that forms the best compromise between the different structure predictions. This is true because similar secondary structures yield edges with a lower weight.

In the final stage of the algorithm, we address the relaxation we have employed by comparing only structures of adjacent sequences. It is reasonable to suggest that a different ordering of the sequences could have produced a different shortest path yielding a different set of proposed structures. To partially compensate for this relaxation the above procedure (stages one to three) is reiterated several times, each with a different input sequence order. Each run results in a new (perhaps overlapping) proposed path. The shortest paths are re-ranked based on the 'Sum-of-Pairs' value. The ranking is performed by going over the n(n−1)2
 MathType@MTEF@5@5@+=feaafiart1ev1aaatCvAUfKttLearuWrP9MDH5MBPbIqV92AaeXatLxBI9gBaebbnrfifHhDYfgasaacH8akY=wiFfYdH8Gipec8Eeeu0xXdbba9frFj0=OqFfea0dXdd9vqai=hGuQ8kuc9pgc9s8qqaq=dirpe0xb9q8qiLsFr0=vr0=vr0dc8meaabaqaciaacaGaaeqabaqabeGadaaakeaadaWcaaqaaiabd6gaUnaabmaabaGaemOBa4MaeyOeI0IaeGymaedacaGLOaGaayzkaaaabaGaeGOmaidaaaaa@33DE@ (where n is the number of sequences) vertices that form the shortest path and summing up the similarity between each of the pairs (i.e. between the two structures each vertex represents) (see Figure 2 (bottom)). After that, we choose the best of the shortest paths proposed (i.e. the path with the smallest Sum-of-Pairs). The program outputs a secondary structure prediction for each of the sequences.

### String and Tree Edit Distance

As each layer of V vertices is compared to its adjacent layer, yielding *O*(*V*^2^) comparisons, the bottleneck of our algorithm is how rapidly it can compare two structures, and how many times it must do so. RNA secondary structure can be represented in several ways from a simple string in bracket notation, to an enriched string representing structural features like loops and bulges, or even as a complex tree structure. Many metrics have been developed to calculate the pairwise distance between RNA secondary structures. M. Höchsmann gives an extensive overview on RNA structure comparison methods in [53]. One common metric is the Hausdorff Distance (HD) which was used by Zuker [54] to filter redundant structures in the Mfold program. The HD between two RNA structures is the maximal distance between each basepair in one structure and its nearest neighbour basepair in the other structure. The linear calculation time of the HD measure makes it useful for coarse (dis)similarity detection. The obvious disadvantage of HD is that it reflects the most extreme dissimilarity between the structures, making it blind to small-but-many dissimilarities.

Another common approach is to represent the RNA secondary structures as a string in bracket notation and to compare them using Needleman-Wunsch's global alignment algorithm [43] which runs in *O*(*N*^2^) time. The major flaw in using this string Edit-Distance (ED) approach is that brackets are not treated as a single unit. A simple example can illustrate the potential problem in using string ED for bracket notation. Consider three strings:

A = ...(...).., B = ...(.....), and C = .(.)......

where a bracket represents matched base and a dot represents an un-matched base.

The ED of {A, B} is 2 mismatches, and the ED of {A, C} is 3. However, in both cases the molecular process is the same; the fourth base has changed its basepair partner, in B the base pairing is between bases 4 and 10 and in C it is between bases 4 and 2.

**Figure 3:**
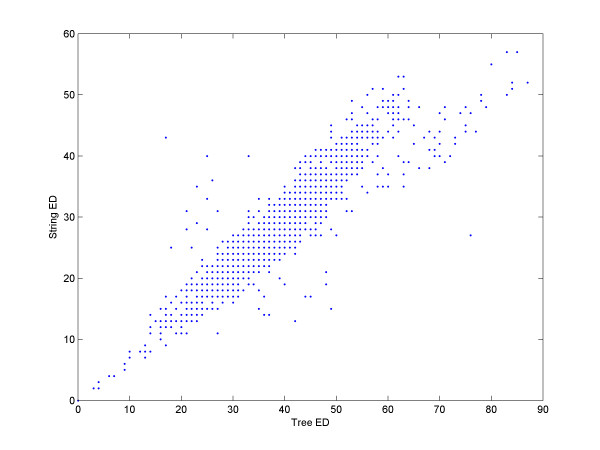
**String Edit-Distance vs. tree Edit-Distance**. The 60 family members of the Lysine family were compared against each other using string and tree ED. The X coordinate of each dot is its tree ED score, and the Y coordinate is its string ED. The correlation coefficient is 0.92, and only a few dots fall far from the main diagonal. Also note that the correlation coefficient of pairs having a tree ED less than or equal to ten is 0.97.

Representing RNA secondary structures as a tree enables a more sensitive comparison of structures, as bases that are paired are treated as an inseparable unit. However, a tree-based approach is considerably slower. Tai [55] was the first to introduce the tree ED metric. Zhang and Shasha [56] suggested an O(T12T22)
 MathType@MTEF@5@5@+=feaafiart1ev1aaatCvAUfKttLearuWrP9MDH5MBPbIqV92AaeXatLxBI9gBaebbnrfifHhDYfgasaacH8akY=wiFfYdH8Gipec8Eeeu0xXdbba9frFj0=OqFfea0dXdd9vqai=hGuQ8kuc9pgc9s8qqaq=dirpe0xb9q8qiLsFr0=vr0=vr0dc8meaabaqaciaacaGaaeqabaqabeGadaaakeaacqWGpbWtdaqadaqaaiabdsfaunaaDaaaleaacqaIXaqmaeaacqaIYaGmaaGccqWGubavdaqhaaWcbaGaeGOmaidabaGaeGOmaidaaaGccaGLOaGaayzkaaaaaa@35F2@ time algorithm. The fastest known algorithm for tree ED is bounded by *O*(*T*^3^) [57]. In our method, we used string ED instead of tree ED in order to improve the run-time for the weight assignment to the edges of the graph. To determine if this heuristic is legitimate, we used the program RNAdistance [16], which is part of the Vienna Package, to calculate tree ED. We calculated the tree ED between all pairs of the 60 members of the Lysine dataset, and compared the results with the simple global alignment distance using weights of one for all edit operations. Figure 3 shows that the tree and string ED are highly correlated. The correlation coefficient between the two datasets is 0.92. Remarkably, we found little difference between the results obtained using the string or tree edit comparisons. Our results strongly indicate that the benefit of tree ED is minimal, especially given the expensive run-time.

### Accelerating string Edit Distance

While string ED is much faster than tree ED, it still requires quadratic run-time. In practice, the run-time can be significantly reduced. As detailed above, each layer of vertices in the DAG is calculated based on the previous (upper) layer. For our application, when calculating a given layer, we need only the best {vertex plus edge} value from the vertices and their edges located in the layer above. Therefore, we initially limit the ED dynamic programming procedure to allow only K (K is a very small number) mistakes (insert, delete, mismatch). This is done easily by filling only the 2*K*-wide-diagonal. Note that this calculation requires time *KN *rather than *N*^2^. If none of the upper vertices has an ED of K or less, K is multiplied by two and the procedure reiterates. In practice, this simple heuristic reduces the run-time without losing accuracy. Moreover, while examining an upper vertex, one can forfeit the ED calculation for this vertex and skip to its neighbour if the value of this vertex plus the minimal expected score of the ED (the previous value of K) is greater (i.e. worse) than the value already obtained for the bottom vertex (for example, if we want to improve the score of a vertex with the current value of 14, there is no point in calculating the ED of vertices with values that exceed 14 nor with vertices of value 12 or more that failed the ED procedure limited to K = 2). In practice, these heuristics allow for a reduction of about 25% in the running time.

### The run-time of RNAspa

We observed that it is not necessary to compare, in the first stage of the algorithm, each of the N sequences (with its V predicted structures) against all the other sequences (with their V predicted structures). Instead, each sequence is compared with only one other, arbitrarily chosen, sequence. We show below that the internal order of the sequences that are to be compared has only a marginal effect on the accuracy of the predicted structures. This observation enabled us to approach this problem by using a linear time (in the number of sequences) graph algorithm technique to search for the shortest path in a DAG.

The algorithm uses two adjustable parameters: the number of suboptimal alignments for each sequence (representing the number of vertices on each level of the DAG), which we can afford to leave quite large, and the number of different sequence orderings or arrangements analyzed before choosing the best alignment.

The algorithm run-time is kept to a reasonable polynomial: *O*(*NL*^3 ^+ *SNV*^2^*L*^2 ^+ *N*^2^*L*^2^) where N is the number of sequences, L is the length of the sequences, S is the number of samples from the sequence's order permutation space, and V is the number of suboptimal structures proposed for each sequence. As N and S are relatively small, the run-time is dominated mostly by *L*^3 ^– the time needed to fold a sequence by RNAsubopt, and *V*^2^*L*^2 ^the time needed to calculate the edges of the graph. Note that because the edges of the graph are only conceptual, they are calculated and immediately used, and therefore need not be maintained in memory.

### RNAspa favours 'correct' structures

**Figure 4:**
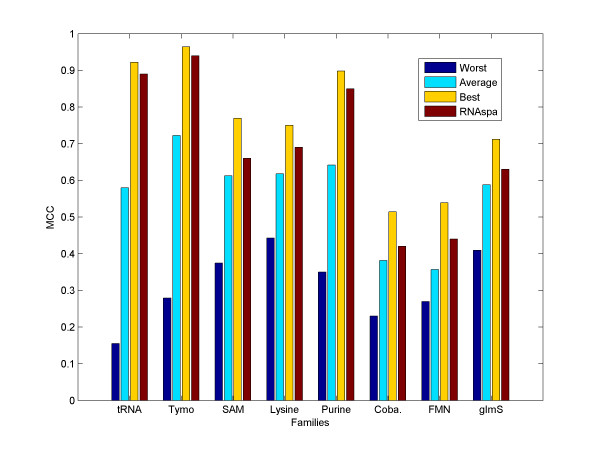
**The average MCC score of the worst/average/best structures suggested by RNAsubopt compared to RNAspa's MCC score**. The average MCC score of the worst/average/best predicted structure in the list of 150 suboptimal structures predicted by RNAsubopt. Note that the performance of RNAspa is much closer to the best score than to the average score.

For each sequence, we took the 150 structure predictions suggested by RNAsubopt and calculated the worst, average, and best MCC scores for these structures. We then compared them with the MCC score of the prediction selected by our RNAspa algorithm. The results, averaged over each family, are shown in Figure 4. The results show that the structures suggested by RNAspa are clearly better than the average of the predicted structures, and in most cases are very close to the best possible structure that is present in the set of RNAsubopt predictions. Thus, in a very reasonable running time, our method is capable of extracting from RNAsubopt the most accurate structures.

### Sampling the permutation space of the sequence order

**Figure 5:**
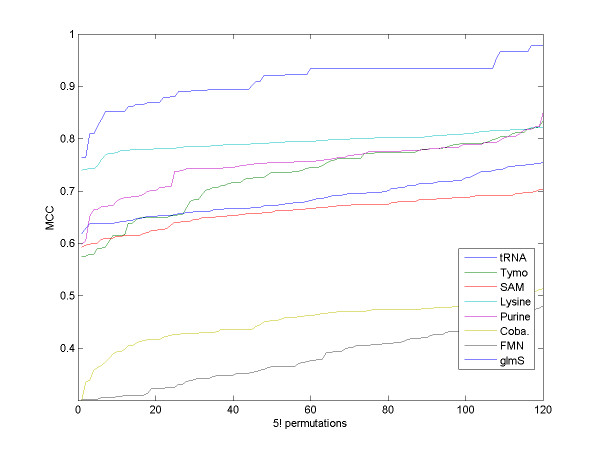
**The influence of sequence order on accuracy**. MCC score of RNAspa over all order permutations of five sequences chosen randomly from each family. The MCC scores are sorted from left to right over the 120 permutations for each family. One can see that the scores increase gradually and that most permutations yield similar results.

**Figure 6:**
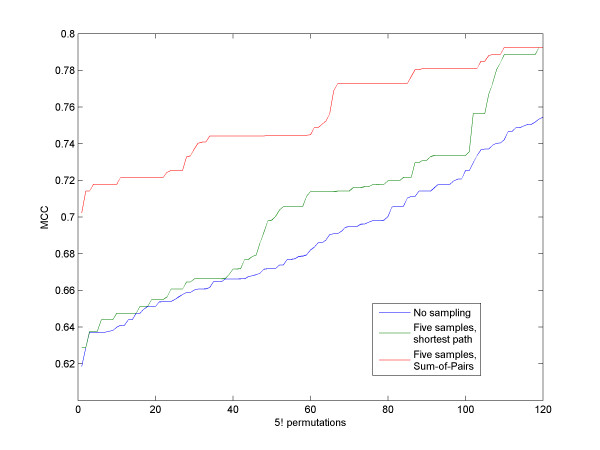
**Shortest path vs. Sum-of-Pairs**. The graph shows the MCC score over 120 (5!) permutations order of the five sequences of the glmS dataset that was used in Table 1. The MCC score is ranked from worst (left) to best (right). The lower (blue) line shows the value achieved by each one of the 120 permutations. The green line shows the increase in accuracy when the best of five different orders was reported. The upper (red) line shows the results where the path was ranked by using the Sum-of-Pairs approach i.e. summing the comparisons between all the pairs that comprise the path. These results clearly show that using the Sum-of-Pairs measure yields better predictions.

We first tested the robustness of our algorithm to the arbitrary order of the sequences chosen. We randomly chose a set of five sequences from each family. We ran RNAspa on all 5! = 120 order permutations to measure the variation of the accuracy level as a function of the sequence order. We used 150 suboptimal structures for each sequence. Figure 5 shows that while some permutations show a significant deviation from the typical MCC score, most permutations exhibit similar results; thus, it is unlikely that a particular randomly selected order will yield the poorest results. Nevertheless, as described above, in order to reduce the probability of choosing an order that will yield a poor prediction, for each run we used several arbitrary fixed orders and calculated the shortest path for each of them. The winning solution was the solution with the lowest Sum-of-Pairs value between all the secondary structures comprising its shortest path. Figure 6 shows that selecting the winning permutation based on the Sum-of-Pairs of the predictions comprising the shortest path is an improvement over using the permutation with the shortest path. The fact that the algorithm is based on calculating paths in linear time, and only in the final stage is a quadratic time Sum-of-Pair score calculated, enables the algorithm to scale, in practice, almost linearly with the number of sequences.

### The effect of various parameters on RNAspa performance

**Figure 7:**
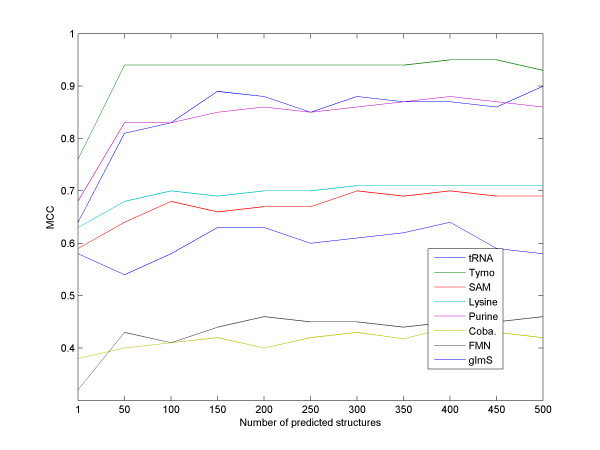
**The influence of the number of suboptimal structures on accuracy**. The performance of the algorithm as a function of the number of suboptimal predictions used as input for our eight datasets. For most datasets, the results improve substantially for the first 50–150 predictions. A further increase in the number of suboptimal structures does not yield much better accuracy. Hence, a value of 150 predictions was chosen as the default for the RNAspa program.

We next measured the effect of our major parameter, the number of suboptimal solutions considered for each sequence, on the performance of RNAspa. Results are shown in Figure 7. As expected, a larger set of suboptimal solutions tested yields better accuracy. However, it seems that about 150 vertices are sufficient, as the accuracy level doesn't increase substantially if the number of vertices is further increased.

**Figure 8:**
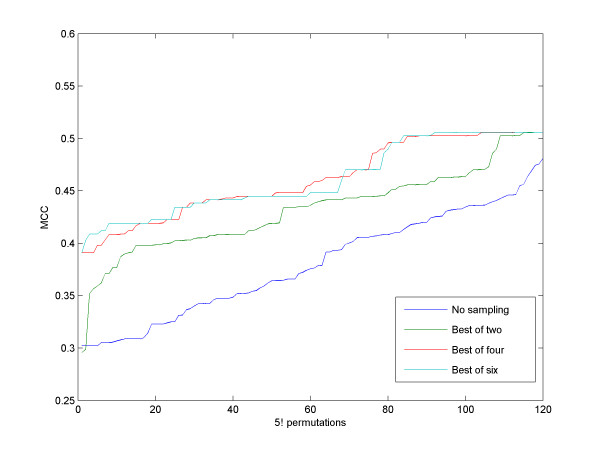
**The influence of sampling on accuracy**. MCC score over 120 (5!) permutations of an FMN dataset. As the number of samples increases, the MCC score is improved. The blue line is the same line as shown for FMN in Figure 3. Note that as the number of samplings increases above four, the improvement diminishes.

The second parameter used by RNAspa is the number of samplings performed on the space of permutations of the order of the sequences. As we illustrated above, comparing the sequences in an arbitrary order is likely to yield an average MCC score. However, our results suggested that performing a small number of samplings on the order permutation space is sufficient to ensure reliable results. On the other hand, it is difficult to identify the optimal order, as the search space is exponential in the number of sequences. In practice, the optimal result offers only a small improvement over the typical performance of the algorithm, while it demands exponential time as there are *N*! possible permutations, where N is the number of sequences. The contribution of the number of samplings to the accuracy level is illustrated in Figure 8. With small sample size, the MCC score improves as the number of samples taken increases, but it reaches limiting returns when the number of samples exceeds four. The diminishing improvement can be explained by the fact that only a small fraction of the order permutations need to be re-sampled in order to significantly improve their score.

**Figure 9:**
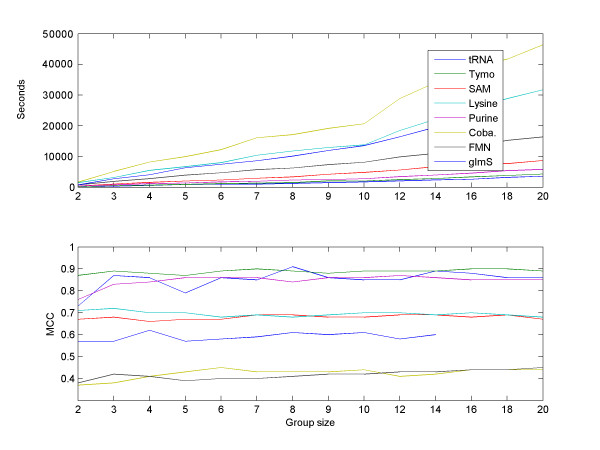
**The influence of the set size on the accuracy and on the running time**. We randomly selected sets of 2–10, 12, 14, 16, 18, 20 sequences from each family. Top: The run-time of the algorithm is roughly linear in the number of sequences. Note that although the Sum-of-Pairs stage is quadratic in the number of sequences, it has only a minor effect on the overall run-time. Bottom: The performance of RNAspa (in terms of the MCC) generally improves as the set size increases from two to about seven or eight. Increasing the size of the sets further, does not improve the performance.

We also checked how the number of sequences in a set influences the accuracy of RNAspa and its running time. As Figure 9 illustrates, the performance (in terms of the MCC of the algorithm) generally improves as the set size increases from 2 to about 7 or 8. For larger sets, the accuracy is not affected by set size. Note that the run-time is close to linear in the number of sequences.

### Comparison with other programs

We compared our method with four state-of-the-art programs: StemLoc (a component of the DART library, version 0.19b) [32,35], pmMulti [9], FoldAlignM 1.0.1 [37], and RNAcast (a component of RNAshapes, version 2.1.1 [38,39]). By default, RNAspa was used with 150 suboptimal structures of each sequence, and 5 samplings of the permutation space. StemLoc was configured with nf = 100. We also ran StemLoc with nf = 1000 but the results did not show a clear improvement and the running time was about 20 fold slower (data not shown). pmMulti, FoldAlignM, and RNAcast were run with their default parameters. The results, based on correlation of the MCC score to the Rfam annotation, which was used as a 'gold standard', are shown in Table 1. In our trials, each the four programs (StemLoc, pmMulti, FoldAlignM, and RNAcast) failed to produce results for at least one of the datasets of the eight families. For two families, Cobalamin and glmS, pmMulti and FoldAlignM failed and could not produce an output due to limitations on sequence length and memory requirements, respectively. RNAcast failed to give a prediction in at least one dataset for seven of the eight families because it couldn't find a common structure. RNAspa was able to handle all the datasets. The performance varied for the different families, but overall, RNAspa performed better than the four other programs. For three out of the eight families, it provided the best MCC scores.

**Figure 10:**
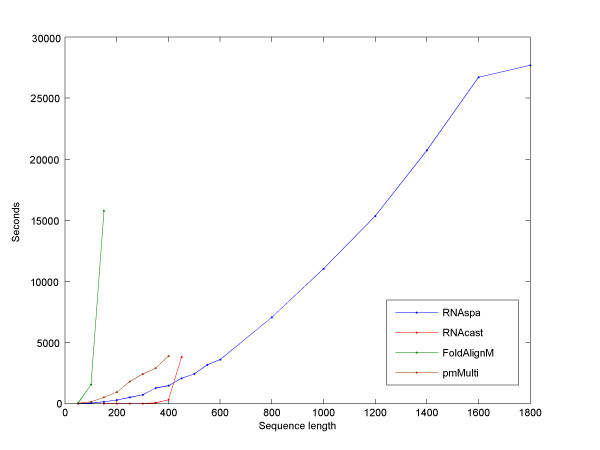
**The influence of length on run-time**. Comparison of the five programs processing increasingly longer windows of a set of five rRNA SSU sequences. Note that RNAspa was run in Boltzmann sampling mode across all sequence lengths. All but RNAspa failed to run on sequences greater than 450 bps due to memory constraints. StemLoc does not appear in the graph because it failed to process sequences of 100 bps or more. As expected, a cubic trendline (not shown) fits RNAspa's curve with the R^2 ^value of 0.9965. RNAspa gave a MCC score of 0.34 for the complete ~1,800 bps long SSU family.

We measured the run-time of the programs. Table 3 shows their performance under the same configuration used in Table 1. We also wanted to explore the relation between the run-time and the length of the sequences in the dataset. We used a set of five SSU rRNA sequences and we measured the run-time for increasingly longer windows. Figure 10 illustrates RNAspa's ability to outperform other programs both in terms of the effect that increasing sequence size has on the runtime and its ability to run on long sequences. All computations were performed on an Intel Xeon 3.0 GHz CPU with 8 GB RAM running Linux.

**Table 3:** Comparison of RNAspa's run-time with that of other methods

Family	Average run-time per sequence in seconds				
	
	RNAspa	StemLoc	pmMulti	FoldAlignM	RNAcast
tRNA	**13**	47	21	549	<1
Tymo	**8**	70	12	93	<1
SAM	**13**	3	18	248	<1
Lysine	**20**	11	69	1076	<1
Purine	**84**	339	135	1518	<1
Cobalamin	**133**	105	no data	no data	<1
FMN	**56**	6	141	5042	<1
glmS	**58**	60	no data	no data	no data

For all datasets, except glmS which did not yield results, the runtime of RNAcast was less than one second. RNAspa always ran faster than pmMulti and FoldAlignM. In four of eight families it ran faster than StemLoc and its overall running time is better than StemLoc's run-time.

The results show that RNAcast and RNAspa, the two non Sankoff-based programs, offer the best combination of performance and runtime. RNAcast was significantly faster. In order to further compare the accuracy of RNAspa and RNAcast, we performed an additional test using the same data set that was initially used to evaluate RNAcast [38]. The results, shown in Table 4, show that while both programs performed well, the accuracy of RNAspa was somewhat higher than that of RNAcast (default parameters) for seven out of ten families. An important difference between RNAspa and RNAcast is that the RNAcast program must be able to find a consensus shape in order to process a dataset, while RNAspa will always return a structural prediction. To evaluate the implications of this difference, we examined the ability of RNAspa and RNAcast to handle a contaminated dataset. Frequently, a set of sequences may be 'contaminated' by one or more sequences that do not actually belong to the same family. We took the 10 families mentioned in Table 4 and randomly picked an additional sequence from another family and added it to the dataset (details of these contaminated datasets can be found in [Additional File 4]). The results in Table 5 illustrate that with a contamination of a single sequence, RNAcast was unable to process six out of the ten families, while RNAspa was able to produce results in all runs.

**Table 4:** Comparison of RNAspa with RNAcast using different RNAcast parameters

Family	Avg. sequence identity	MCC				
		
		RNAspa	RNAcast			
			-t 5 -c 10 (default)	-t 5 -c 20	-t 3 -c 10	-t 3 -c 20
lin4	66%	**0.86**	0.73	0.73	no data	0.72
tRNA	50%	**0.81**	0.42	0.42	no data	0.65
5S RNA	59%	**0.58**	0.62	0.62	no data	0.84
U2	78%	**0.74**	0.72	0.72	0.69	0.69
S box riboswitch	67%	**0.75**	0.69	0.69	0.78	0.78
SRP RNA	52%	**0.95**	0.99	0.99	0.94	0.94
IRES	66%	**0.97**	0.93	0.93	0.90	0.90
Purine riboswitch	56%	**0.93**	0.78	0.78	0.81	0.81
U1	65%	**0.67**	0.72	0.72	no data	0.76
U12	83%	**0.76**	0.50	0.50	no data	0.68

RNAcast was run using four different configurations. RNAcast parameters that were used: -t sets the shape type (default: 5). Shape type defines the level of abstraction from most accurate to most abstract (1–5). -c sets the energy range (default: 10%). For seven out the ten families RNAspa gave better results. See [Additional File 7] for raw data.

**Table 5:** Comparison of RNAspa with RNAcast on contaminated datasets

Family	MCC	
	
	RNAspa	RNAcast
lin4	**0.86**	no data
tRNA	**0.80**	0.42
5S RNA	**0.51**	no data
U2	**0.72**	no data
S box riboswitch	**0.77**	0.82
SRP RNA	**0.93**	0.78
IRES	**1.00**	no data
Purine riboswitch	**0.90**	0.78
U1	**0.61**	no data
U12	**0.83**	no data

Contaminated datasets were constructed by adding a single randomly picked sequence from a different family to each family tested. RNAspa was able to produce good results for all families while RNAcast was unable to find a consensus structure for six of the ten families in a contaminated dataset. See [Additional File 8] for raw data.

### Robustness of RNAspa to contaminated datasets

**Figure 11:**
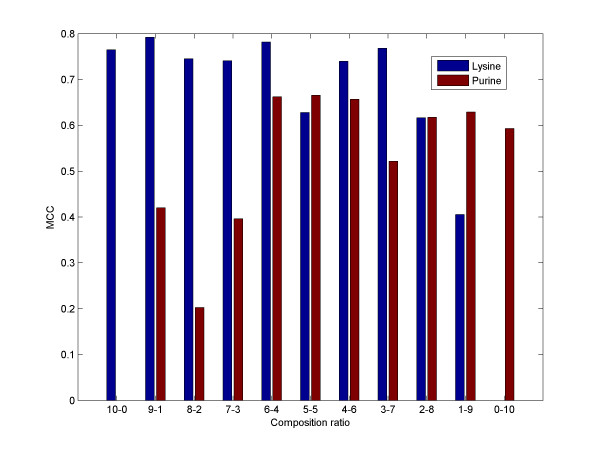
**An example with a contaminated dataset**. The leftmost bar represents the MCC score of a set of ten Purine sequences. The rightmost bar represents a set of ten sequences from the Lysine family. Towards the middle of the graph, the sets become more and more mixed. Note that with increasing contamination, the results tend to deteriorate, but in general the method is robust to low levels of contamination.

We further investigated the extent to which the performance of RNAspa can withstand the effects of contaminated data. Specifically in our algorithm, a sequence that has a very different set of potential suboptimal structures would break the path into two detached components. As one would expect, a contaminated set reduces the performance of the algorithm. However, RNAsubopt's worst MCC score serves as a 'safety net' in these cases. Figure 11 shows the performance of the algorithm when two different datasets (Purine and Lysine) were mixed together in varying proportions starting with a set of ten Lysine sequences, followed by a set of nine Lysine and one Purine, then eight Lysine and two Purine, and so on. The results show that our method is quite robust to this kind of contamination, although, as expected, as the number of sequences that do no belong to the family increases, there is a negative effect on the performance.

## Discussion

To illustrate the advantage of the shortest path approach, we review two alternative straightforward approaches for the task of comparative prediction of secondary structures, using suboptimal predictions. It is easy to see that an optimal solution would involve using weighted edges to connect each of the vertices of the DAG with the vertices on the other layers and then finding the Minimum Edge-Weighted Clique (MEWC) of size of N – the number of sequences. The run-time would be exponential, as MEWC is an NP-hard problem [58], although heuristic approaches can be used to reduce the run-time in practice. The second approach is based on a polynomial time greedy bottom-up UPGMA-like algorithm. The idea is to find the most 'similar' pair of sequences (i.e. the two sequences that share the most similar common structure) and then 'merge' the pair and reiterate the procedure. This algorithm demands quadratic time in the number of sequences, making it feasible. However, there are quite a few pitfalls of the latter algorithm. The merging process of two structures might favour a third structure that does not resemble either of the original two. Moreover, the initial pairs, although having the best score, might prove a poor starting point leading to a weak overall solution.

RNAspa, unlike the latter algorithm, uses a different relaxation. The time complexity is reduced from exponential to linear (in the number of sequences) by a heuristic that imposes order on the list of sequences. As the order we impose is arbitrary, and as each different order yields a different solution, the algorithm is not guaranteed to find the optimal solution. However, we have demonstrated (Figure 5) that by sampling a relatively small number of arbitrary orders, an order close to the optimal can be found. The process of finding the shortest path gives an equal weight to each potential structure, yielding a solution that is the best compromise for all. Of course, this advantage comes with a price; each sequence (with its predicted structures) is compared with only one other sequence. In this context, it is important to note, that the comparison process of sequences A to B and B to C, retains a great deal of information on the relation between A to C. For two edges between *A*_*i *_to *B*_*j *_and *B*_*j *_to *C*_*k *_we can be sure that an edge between *A*_*i *_and *C*_*k *_will be less or equal in weight to the two edges. Therefore, the weight of the shortest path is a lower bound of the overall similarity. The process of finding the shortest path indirectly harnesses these transitive relations.

Why is the string ED sufficient for RNAspa? It is important to remember that only similar structures are of interest. We have shown in Figure 3 a correlation of 0.92 between tree ED and string ED. We also showed in that figure that the correlation between the results that had tree ED less than or equal to ten, is even better: 0.97. We can explain this very high correlation by suggesting that similar structures have, in many cases, the same topology (i.e. the difference is only in the lengths of the stems, loops, and gaps). In this case, tree and string ED are highly correlated. When the topology is different, in many cases, stems (which are usually three bps or more in length) need to be relocated or created; therefore, there are many letters/nodes that are involved in the 'correction' procedure, thus the overall similarity is poor, and usually not relevant.

RNAcast and RNAspa follow the same logic of investigating the space of suboptimal structures. RNAcast is extremely fast and thus should be used in cases when speed is important. On the other hand, RNAspa performed a little better in our benchmarks. However, the greater advantage of RNAspa over RNAcast is its ability to address 'contaminated' data, i.e. situations in which not all sequences share the common structure, as described in Table 5. Note that this is not only a problem with an artificial experiment. Looking at Table 1, one can notice that RNAcast failed several times to produce an output for several datasets of the same family of ncRNA, probably due to the fact that it did not find a common shape for all sequences. Another advantage of RNAspa is its ability to address longer sequences. Using SSU rRNA as an example, we were able to run the program on sequences of length 1800 bps, where as RNAcast was not able, at least in its current public version, to address such lengths.

Inspired by the power of abstraction of RNA structures that was demonstrates by RNAcast, we suggest an application that might help in finding unknown ncRNA families hiding within a large set of ncRNA candidates [37,44]. Similar to RNAcast, many suboptimal abstract shapes are can be generated for each sequence. Then, the abstract shapes of all the sequences in the set can be sorted, clustering together different sequences that share the same abstract shape. Each set of sequences sharing the same abstract shape is a potential ncRNA family. Initial results produced by a prototype program that we developed confirmed the speed and feasibility of this direction.

An interesting application of algorithms like RNAcast and RNAspa that are based on looking for common structures in the pile of suboptimal structures might be in predicting the secondary structure of riboswitches [59]. Those are a class of ncRNA molecules that can adapt more than one possible structure, usually upon interaction with different ligands. As suggested in [58], a set of riboswitches can be identified if it has two consistent suboptimal structures, each for a different conformation of the riboswitch, that are present for all (or most) of the sequences. RNAspa can be modified to find such cases by looking for the two shortest paths in the graph. We note however that this is not trivial as the second best path is likely to be a variation of the first path rather than a totally different path. One possibility to address this problem is to eliminate from the graph all the nodes that participate in the first path and other similar nodes before looking for the second path. Similar issues have been addressed when looking into suboptimal sequence alignments [60].

## Conclusion

We note that current RNA secondary structure prediction algorithms are still far away from producing consistent satisfactory predictions. However, within the set of currently available programs our approach is fast, simple and on average performs better than other secondary structure prediction methods. RNAspa relies heavily on the long-standing work of the Vienna Package team, their knowledge and available applications, and further extends its performance. We intend to use our tool as a component of a wider clustering algorithm to identify novel families of ncRNA from a set of ncRNA candidates.

## Availability and requirements

Project name: RNAspa

Supplementary information: 

Operating systems: Unix, Linux.

Programming language: C/C++.

Restrictions on non-academic use: none.

## Authors' contributions

YH developed the core idea of the algorithm and implemented it. TD tested the implementation extensively and helped in comparing the performance of RNAspa to that of other programs. SM provided advice about the biological applications of the algorithm, and RU suggested approaches to evaluate the algorithm and demonstrate its performance. All authors read and approved the final manuscript.

### Note added in proof

The FoldalignM program offers two versions, one is based on Foldalign matrices and   the other on McCaskill matrices. We unfortunately arbitrarily have chosen to use the   Foldalign based version. We later became aware by the authors that the version  that uses the McCaskill matrices runs much faster. Thus, the results presented here   reflect only the performance of the slower version and not the real performance of the   FoldalignM method.

## Supplementary Material

Additional file 1 Sets of ten sequences. Random non-overlapping sets of ten sequences. Click here for file

Additional file 2 Sets of five sequences. Random non-overlapping sets of five sequences. Click here for file

Additional file 3 Sequences and structures. The eight families and the rRNA SSUs family with their Rfam annotated structure. Click here for file

Additional file 4 Contaminated sets. The dataset used from the supplementary data of: Jens Reeder and Robert Giegerich: Consensus Shapes: An Alternative to the Sankoff Algorithm for RNA Consensus Structure Prediction. Bioinformatics 2005 21(17): 3516–3523. A contaminated version of each family was constructed by adding a randomly chosen sequence from a different family to each family. The first sequence of each set is the impostor. Click here for file

Additional file 5 Table 1 raw data. The predicted structures of RNAspa whose MCC scores is reported in Table 1. Each file contains a concatenation of the output RNAspa gave to all the group of ten sequences for each family. Click here for file

Additional file 6 Table 2 raw data. The predicted structures of RNAspa whose MCC scores is reported in Table 2. Each of the eight families was compared running both modes. Click here for file

Additional file 7 Table 4 raw data. The predicted structures of RNAspa whose MCC scores is reported in Table 4. The dataset used from the supplementary data of: Jens Reeder and Robert Giegerich: Consensus Shapes: An Alternative to the Sankoff Algorithm for RNA Consensus Structure Prediction. Bioinformatics 2005 21(17): 3516–3523. Click here for file

Additional file 8 Table 5 raw data. The predicted structures of RNAspa whose MCC scores is reported in Table 5. Click here for file

## References

[B1] Huang ZP, Zhou H, He HL, Chen CL, Liang D, Qu LH (2005). Genome-wide analyses of two families of snoRNA genes from Drosophila melanogaster, demonstrating the extensive utilization of introns for coding of snoRNAs. RNA.

[B2] Carter RJ, Dubchak I, Holbrook SR (2001). A computational approach to identify genes for functional RNAs in genomic sequences. Nucleic Acids Res.

[B3] Chen S, Lesnik EA, Hall TA, Sampath R, Griffey RH, Ecker DJ, Blyn LB (2002). A bioinformatics based approach to discover small RNA genes in the Escherichia coli genome. Biosystems.

[B4] Tjaden B, Saxena RM, Stolyar S, Haynor DR, Kolker E, Rosenow C (2002). Transcriptome analysis of Escherichia coli using high-density oligonucleotide probe arrays. Nucleic Acids Res.

[B5] Erdmann VA, Barciszewska MZ, Hochberg A, de Groot N, Barciszewski J (2001). Regulatory RNAs. Cell Mol Life Sci.

[B6] Kiss T (2002). Small nucleolar RNAs: an abundant group of noncoding RNAs with diverse cellular functions. Cell.

[B7] Mattick JS (2003). Challenging the dogma: the hidden layer of non-protein-coding RNAs in complex organisms. Bioessays.

[B8] Mattick JS, Makunin IV (2006). Non-coding RNA. Hum Mol Genet.

[B9] Hofacker IL, Bernhart SH, Stadler PF (2004). Alignment of RNA base pairing probability matrices. Bioinformatics.

[B10] Rivas E, Eddy SR (2000). Secondary structure alone is generally not statistically significant for the detection of noncoding RNAs. Bioinformatics.

[B11] Liang XH, Uliel S, Hury A, Barth S, Doniger T, Unger R, Michaeli S (2005). A genome-wide analysis of C/D and H/ACA-like small nucleolar RNAs in Trypanosoma brucei reveals a trypanosome-specific pattern of rRNA modification. RNA.

[B12] Washietl S, Hofacker IL, Stadler PF (2005). Fast and reliable prediction of noncoding RNAs. Proc Natl Acad Sci U S A.

[B13] Mathews DH, Sabina J, Zuker M, Turner DH (1999). Expanded sequence dependence of thermodynamic parameters improves prediction of RNA secondary structure. J Mol Biol.

[B14] Zuker M (2003). Mfold web server for nucleic acid folding and hybridization prediction. Nucleic Acids Res.

[B15] Hofacker IL (2003). Vienna RNA secondary structure server. Nucleic Acids Res.

[B16] Hofacker IL, Fontana W, Stadler PF, Bonhoeffer S, Tacker M, Schuster P (1994). Fast folding and comparison of RNA secondary structures. Monatshefte f Chemie.

[B17] Zuker M, Stiegler P (1981). Optimal Computer Folding of Large Rna Sequences Using Thermodynamics and Auxiliary Information. Nucleic Acids Research.

[B18] Nussinov R, Pieczenik G, Griggs JR, Kleitman DJ (1978). Algorithms for Loop Matchings. Siam Journal on Applied Mathematics.

[B19] Serra MJ, Turner DH (1995). Predicting thermodynamic properties of RNA. Methods Enzymol.

[B20] Reeder J, Hochsmann M, Rehmsmeier M, Voss B, Giegerich R (2006). Beyond Mfold: recent advances in RNA bioinformatics. J Biotechnol.

[B21] Wuchty S, Fontana W, Hofacker IL, Schuster P (1999). Complete suboptimal folding of RNA and the stability of secondary structures. Biopolymers.

[B22] Hofacker IL, Fekete M, Stadler PF (2002). Secondary structure prediction for aligned RNA sequences. J Mol Biol.

[B23] Knight R, Birmingham A, Yarus M (2004). BayesFold: rational 2 degrees folds that combine thermodynamic, covariation, and chemical data for aligned RNA sequences. RNA.

[B24] Luck R, Graf S, Steger G (1999). ConStruct: a tool for thermodynamic controlled prediction of conserved secondary structure. Nucleic Acids Res.

[B25] Voss B (2006). Structural analysis of aligned RNAs. Nucleic Acids Res.

[B26] Siebert S, Backofen R (2005). MARNA: multiple alignment and consensus structure prediction of RNAs based on sequence structure comparisons. Bioinformatics.

[B27] Hochsmann M, Voss B, Giegerich R (2004). Pure multiple RNA secondary structure alignments: a progressive profile approach. IEEE/ACM Trans Comput Biol Bioinform.

[B28] Mathews DH, Turner DH (2002). Dynalign: an algorithm for finding the secondary structure common to two RNA sequences. J Mol Biol.

[B29] Tabei Y, Tsuda K, Kin T, Asai K (2006). SCARNA: fast and accurate structural alignment of RNA sequences by matching fixed-length stem fragments. Bioinformatics.

[B30] Chen JH, Le SY, Maizel JV (2000). Prediction of common secondary structures of RNAs: a genetic algorithm approach. Nucleic Acids Res.

[B31] Hamada M, Tsuda K, Kudo T, Kin T, Asai K (2006). Mining frequent stem patterns from unaligned RNA sequences. Bioinformatics.

[B32] Holmes I, Rubin GM (2002). Pairwise RNA structure comparison with stochastic context-free grammars. Pac Symp Biocomput.

[B33] Sankoff D (1985). Simultaneous solution of the RNA folding, alignment and protosequence problems. SIAM Journal on Applied Mathematics.

[B34] Gardner PP, Giegerich R (2004). A comprehensive comparison of comparative RNA structure prediction approaches. BMC Bioinformatics.

[B35] Holmes I (2005). Accelerated probabilistic inference of RNA structure evolution. BMC Bioinformatics.

[B36] McCaskill JS (1990). The equilibrium partition function and base pair binding probabilities for RNA secondary structure. Biopolymers.

[B37] Torarinsson E, Havgaard JH, Gorodkin J (2007). Multiple structural alignment and clustering of RNA sequences. Bioinformatics.

[B38] Reeder J, Giegerich R (2005). Consensus shapes: an alternative to the Sankoff algorithm for RNA consensus structure prediction. Bioinformatics.

[B39] Steffen P, Voss B, Rehmsmeier M, Reeder J, Giegerich R (2006). RNAshapes: an integrated RNA analysis package based on abstract shapes. Bioinformatics.

[B40] Gardner PP, Wilm A, Washietl S (2005). A benchmark of multiple sequence alignment programs upon structural RNAs. Nucleic Acids Res.

[B41] Wilm A, Mainz I, Steger G (2006). An enhanced RNA alignment benchmark for sequence alignment programs. Algorithms Mol Biol.

[B42] Bindewald E, Shapiro BA (2006). RNA secondary structure prediction from sequence alignments using a network of k-nearest neighbor classifiers. RNA.

[B43] Needleman SB, Wunsch CD (1970). A general method applicable to the search for similarities in the amino acid sequence of two proteins. J Mol Biol.

[B44] Will S, Reiche K, Hofacker IL, Stadler PF, Backofen R (2007). Inferring Noncoding RNA Families and Classes by Means of Genome-Scale Structure-Based Clustering. PLoS Comput Biol.

[B45] Griffiths-Jones S, Bateman A, Marshall M, Khanna A, Eddy SR (2003). Rfam: an RNA family database. Nucleic Acids Res.

[B46] (2007). RNA families database of alignments and CMs. http://www.sanger.ac.uk/Software/Rfam/.

[B47] (2007). Gutell Lab CRW Site. http://www.rna.ccbb.utexas.edu/.

[B48] Durbin R, Eddy S, Krogh A, Mitchison G (1998). Biological SequenceAnalysis: Probabilistic Models of Proteins and Nucleic Acids.

[B49] Eddy SR, Durbin R (1994). RNA sequence analysis using covariance models. Nucleic Acids Res.

[B50] Eddy SR (2002). A memory-efficient dynamic programming algorithm for optimal alignment of a sequence to an RNA secondary structure. BMC Bioinformatics.

[B51] Gorodkin J, Stricklin SL, Stormo GD (2001). Discovering common stem-loop motifs in unaligned RNA sequences. Nucleic Acids Res.

[B52] Hu YJ (2002). Prediction of consensus structural motifs in a family of coregulated RNA sequences. Nucleic Acids Res.

[B53] M H (2005). The Tree Alignment Model: Algorithms, Implementations and Applications for the Analysis of RNA Secondary Structures.

[B54] Zuker M (1989). On finding all suboptimal foldings of an RNA molecule. Science.

[B55] Tai KC (1979). Tree-To-Tree Correction Problem. Journal of the Acm.

[B56] Zhang KZ, Shasha D (1989). Simple Fast Algorithms for the Editing Distance Between Trees and Related Problems. Siam Journal on Computing.

[B57] Demaine ED, Mozes S, Rossman B, Weimann B (2006). An O(n3)-time algorithm for tree edit distance. Arxiv preprint cs DS/0604037.

[B58] Macambira EM, de Souza CC (2000). The edge-weighted clique problem: Valid inequalities, facets and polyhedral computations. European Journal of Operational Research.

[B59] Nudler E (2006). Flipping riboswitches. Cell.

[B60] Vingron M (1996). Near-optimal sequence alignment. Curr Opin Struct Biol.

